# Editorial: Innovative Approaches to Tackle Atrial Fibrillation: From Bench to Bedside

**DOI:** 10.3389/fcvm.2020.566239

**Published:** 2020-10-09

**Authors:** Xun Ai, Bianca J. J. M. Brundel

**Affiliations:** ^1^Department of Physiology and Biophysics, Rush University Medical Center, Chicago, IL, United States; ^2^Department of Physiology, Amsterdam University Medical Centers (UMC), Vrije Universiteit, Amsterdam Cardiovascular Sciences, Amsterdam, Netherlands

**Keywords:** atrial fibrillation, proteostasis, DNA damage, genetics, atrial cardiomyopathy, stroke, optical genetics, sex differences

**Graphical Abstract:**
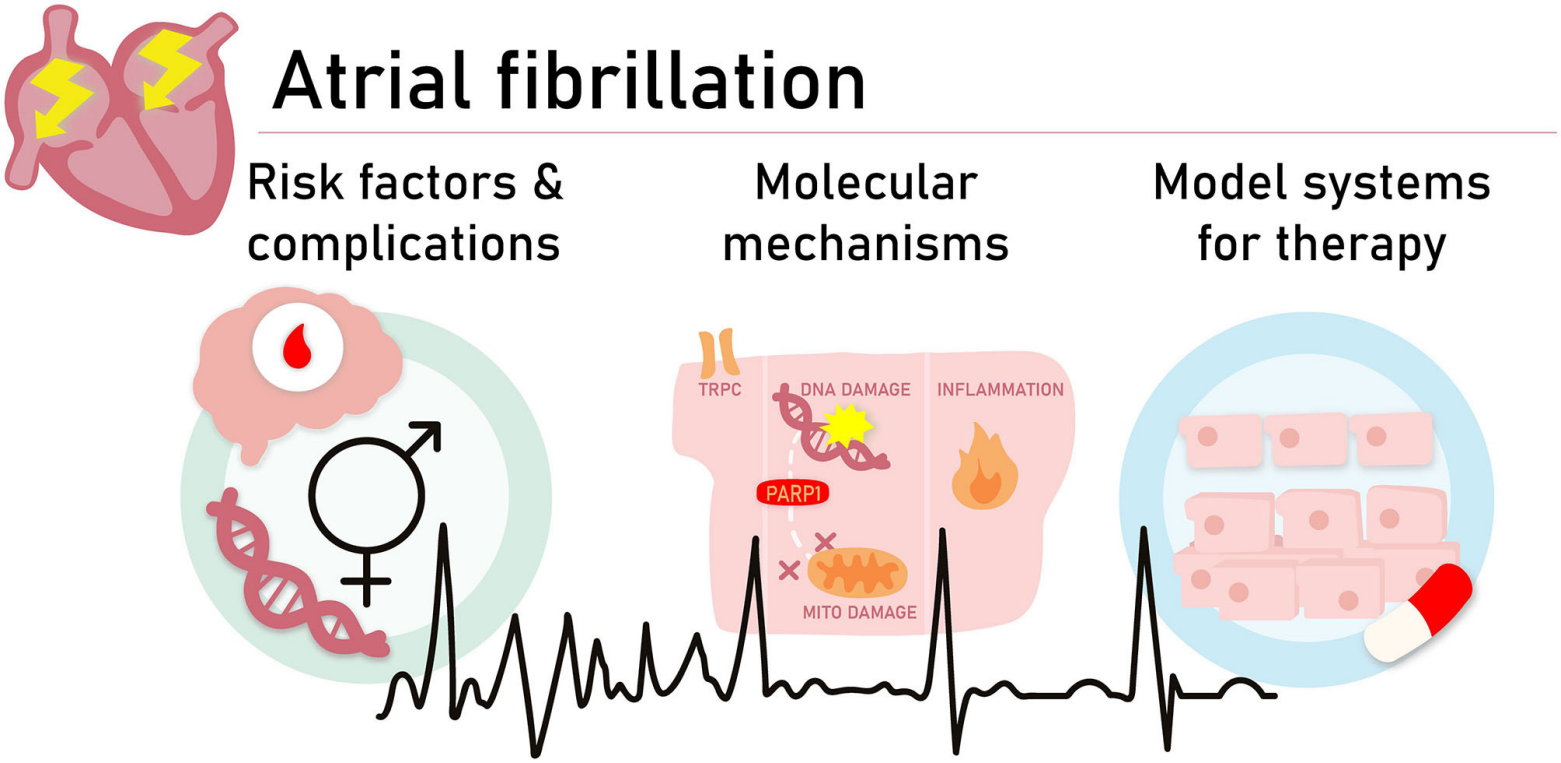
To obtain a better understanding of the molecular pathways driving AF and the development of novel treatment strategies, we need to investigate several aspects and unmet needs related to AF onset and progression. Aspects and unmet needs include (1) improved insights in risk factors contributing to AF onset and pathways which increase complications related to AF, such as stroke, (2) identification of molecular pathways which drive AF onset, and (3) the development of 2D and 3D experimental AF model systems to dissect pathways and test novel treatment options.

Atrial fibrillation (AF), the most common progressive cardiac rhythm disorder, is present in 2–3% of the Western population and associated with common risk factors, such as advanced age, hypertension, diabetes, and obesity (1). As AF may result in serious complications, including stroke and heart failure, this arrhythmia induces a tremendous economic burden for the individual and society (1). Due to its progressive nature, patients often undergo transition from paroxysmal AF to persistent and longstanding-persistent AF. Importantly, failure rates of AF therapy in persistent and long-standing persistent AF are high (1).

Although AF was originally described as an “electrical” disease related to changes in atrial refractoriness, various recent studies reveal that AF induces atrial molecular remodeling, which, in turn, may impair electrical activation and conduction of the atria (“electropathology”) (2, 3). This new concept of AF explains why the current drug therapies directed at refractoriness show limited efficacy and indicates that it is important to direct research at uncovering novel risk factors and (molecular) pathways driving AF, in order to develop more mechanism-related effective AF therapies. Recently discovered new pathways is AF include inflammation and proteostasis (4), and transcriptional networks (5), but more research is required to complete the picture.

To obtain a better understanding of the molecular pathways driving AF and the development of novel treatment strategies, we need to investigate several aspects and unmet needs related to AF onset and progression (see Graphical Abstract).

First of all, more (epidemiological) research is required to investigate the role of sex differences in AF promotion and responses to therapy. The contribution of Kavousi is directed at identification of differences between sexes. Evidence already indicate an association between sex difference and the presence of various risk factors of AF, including diabetes mellitus, hypertension, and dysglycemia in combination with AF. Less evidence is available on women-specific conditions, such as pregnancy, pregnancy-related complications, number of children as potential risk factors for AF development. More detailed knowledge on sex-related risk factors for AF is required to better understand the molecular mechanisms driving AF and to develop potential sex-related treatment. Hereto, large-scale epidemiological and genome-wide association studies may be utilized.

A major complication associated with AF is stroke. The mechanisms of thromboembolism in AF are complex. Evidence points to a role of three criteria of Virchow's triad in AF: abnormal stasis of blood, endothelial damage, and hypercoagulability. That stroke is still a major issue in patients with AF may be due to the presence of atrial hypocontractility in these patients. Detailed knowledge on the molecular mechanism for hypocontractility is lacking. Darlington and McCauley provide molecular and clinical evidence for atrial cardiomyopathy to underlie hypocontractility and as such stroke. This is a new concept which suggests that treatment directed at prevention of and/or recovery from hypocontractility may attenuate the risk of stroke in AF patients (Darlington and McCauley).

It is well-known that about 30% of the AF patients present without any of the common risk factors. This observation suggests a potential role for a genetic mutation to contribute to AF. Ragab et al. highlight the findings of genetic AF epidemiological studies, describe the role of both rare and common genetic variants as well as their clinical and therapeutic implications. It is important to increase awareness on genetic AF in the clinic and investigate more optimal treatment strategies in mutation carriers.

Another important aspect included in this special issue is the description of molecular mechanisms driving AF. Research indicates that transient receptor potential canonical (TRPC) channels play a role in regulating the function of cardiomyocytes under normal and stressful conditions, such as during AF. For the development of novel drugs against AF, unraveling of the molecular mechanisms regulating these TRPC channels in AF may provide new concepts for the design of compounds (Wen et al.) Besides the electrical remodeling, Ramos and Brundel highlight the role of a novel pathomechanism in AF: oxidative DNA damage-induced poly-ADP-ribose polymerase 1 (PARP1) activation, which results in nicotinamide adenine dinucleotide (NAD^+^) depletion and consequently electrical and contractile dysfunction. So far, DNA damage was found to be associated with cardiac diseases, such as mutation-induced cardiomyopathy and peripartum cardiomyopathy. The role of DNA damage, PARP1 activation, and NAD^+^ depletion opens opportunities for novel pharmacological treatment strategies. Since several drugs within this pathway have been marketed for other indications, a clinical trial in AF patients seems within reach. In addition to DNA damage, inflammation has been shown to drive AF. The review of Zhou and Dudley discusses the role of inflammation in AF onset and progression of AF. Key modulators within the inflammation pathway have been identified as druggable targets, indicating that the next step is mounting of a clinical study in AF patients.

To study novel pathophysiological pathways involved in AF onset and progression, innovative experimental model systems for AF are required. Hereto, monolayers of atrial cardiomyocyte may be used to mimic the atrial myocardium. This approach enables rapid genetic and pharmacological interventions and therefore has been already successfully utilized to elucidate molecular mechanisms underlying AF. In the review of van Gorp et al. an overview of the currently available two- and three-dimensional multicellular atrial cardiomyocyte systems for AF research is discussed. These systems include primary and pluripotent stem cell-derived atrial-like cardiomyocytes and (conditionally) immortalized atrial cardiomyocytes. Each system has its own strengths and weaknesses, but in general, these systems are essential to obtain detailed knowledge on molecular mechanisms driving AF and testing of compounds in a (semi) high-throughput fashion.

In summary, this special issue contains a collection of reviews covering the latest exciting findings and innovative approaches in AF research, including large scale epidemiological and genetic AF screening studies, advancement in the underlying molecular mechanisms of AF substrate remodeling as well as development of experimental model systems. These recently made progresses in AF studies enable broadening of our knowledge on novel mechanisms driving AF onset and progression and to facilitate the identification of novel druggable targets. It is clear that implementation of new research findings for the design of novel diagnostic tools and (pharmacological) treatment strategies to tackle AF is the next step.

## Author Contributions

XA and BB were guest editors of the special issue Innovative approaches to Tackle Atrial Fibrillation, from bench to bedside. All authors contributed to the article and approved the submitted version.

## Conflict of Interest

The authors declare that the research was conducted in the absence of any commercial or financial relationships that could be construed as a potential conflict of interest.
